# Supplementary figures and images for: Firing-rate based network modeling of the dLGN circuit: Effects of cortical feedback on spatiotemporal response properties of relay cells
Source: PLoS Comput Biol. 2018 May 17;14(5):e1006156. doi: 10.1371/journal.pcbi.1006156 (PMC5976212; doi:10.1371/journal.pcbi.1006156)

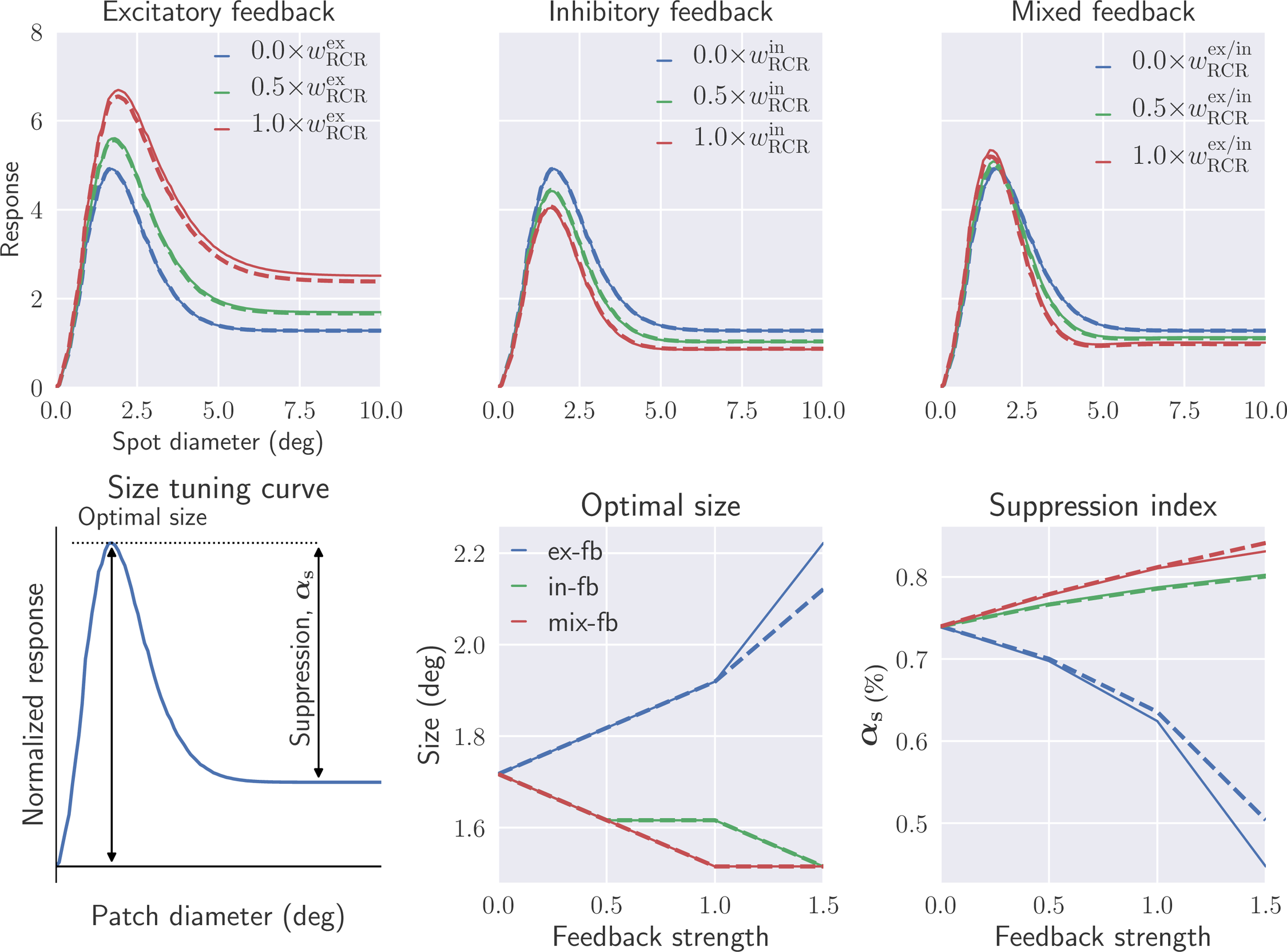

Supplement: S1 Fig — Top row: predicted area-response curves of relay cells for different arrangements of cortical feedback. Excitatory (left) and inhibitory feedback (center): solid lines and dashed lines correspond to short-delay (5 ms) and long-delay (30 ms) feedback, respectively. Mixed feedback: solid lines correspond to delayed inhibition (ΔRCRex=5 ms, ΔRCRin=30 ms), while dashed lines correspond to delayed excitation (ΔRCRex=30 ms, ΔRCRin=5 ms). Bottom row: optimal size and suppression index (αs) are shown as a function of cortical feedback weight for different feedback configurations with different delays. Line styles correspond to different delays as described above. The values on the x-axis represent factors multiplied with the default values for wRCRin and wRCRex listed in Table 1. Default values for fixed parameters are also listed in this table. (TIF) [file pcbi.1006156.s001.tif]
